# Association between angiotensin-converting enzyme inhibitors and the risk of lung cancer: a systematic review and meta-analysis

**DOI:** 10.1038/s41416-022-02029-5

**Published:** 2022-11-17

**Authors:** Zhenchao Wu, Taikang Yao, Zilu Wang, Beibei Liu, Nan Wu, Ming Lu, Ning Shen

**Affiliations:** 1 Department of Pulmonary and Critical Care Medicine, Peking University Third Hospital, 100191 Beijing, P. R. China; 2 Peking University Health Science Center, Peking University, 100191 Beijing, P. R. China

**Keywords:** Lung cancer, Lung cancer

## Abstract

**Background:**

The association between the use of angiotensin-converting enzyme inhibitors (ACEIs) and lung cancer risk remains controversial. This study evaluated the association between the use of ACEIs and lung cancer risk.

**Methods:**

Records from five databases were searched from inception to 26 January 2022. Clinical studies involving persons aged ≥18 years with at least one year of follow-up and reporting adverse events, including lung cancer, were recorded with separate outcome reports supplied for the ACEIs and control groups. Data were extracted independently by three authors and pooled using a random-effects model. The primary outcome was lung cancer development. Odds ratios (ORs) with 95% confidence intervals (CIs) and lung cancer-related morbidity were calculated.

**Results:**

Of 2400 records screened, 13,061,226 patients were included from seven cohort studies and four case–control studies. Pooled results showed that ACEIs use was linked to increased lung cancer risk (OR 1.19, 95% CI 1.05–1.36; *P* = 0.008), with high heterogeneity (*I*^2^ = 98%).

**Conclusions:**

ACEI usage is a greater risk factor for lung carcinogenesis than angiotensin receptor blocker use, especially in Asian patients. Further randomised controlled trials are needed to confirm the causal association between the use of ACEIs and lung cancer risk.

## Background

Angiotensin-converting enzyme inhibitors (ACEIs) are widely prescribed as first-line drugs in chronic diseases that require long-term treatment and management for patients with hypertension and congestive heart failure, especially for those with chronic kidney disease (CKD) or diabetes mellitus (DM) [1]. Captopril, the first orally active ACEI, became available in the late 1970s [2]. Since then, pharmaceutical companies have developed >15 ACEI products that have been approved by the United States Food and Drug Administration.

Based on decades of clinical observations on the use of ACEIs, a series of adverse reactions have attracted increasing attention. Notably, respiratory adverse effects have persisted as nuisance adverse events with the use of ACEIs [3], of which dry cough is a major complication [4]. Some studies have suggested that the use of ACEIs is associated significantly with the risk of airway obstructive symptoms and a worsening risk of bronchoconstriction or asthma [5, 6]. Preclinical carcinogenicity animal studies have shown negative results; however, some studies have reported that ACEIs participate in cellular proliferation, angiogenesis and tumour progression [7, 8]. Randomised controlled trials (RCTs) conducted decades ago mainly evaluated the effect of ACEIs on cardiovascular and renal endpoints, and the risk of cancer was not included [9–11]. Recently, some studies have indicated that patients receiving ACEIs develop lung cancer [12–14], whereas other studies have not suggested that the use of ACEIs is related to such risk [15–17]. These contradictory findings have raised doubts among clinicians and caused concern among patients.

One previous meta-analysis [18] involving 13 studies (*n* = 458,686 ACEI users) reported no significant association between the use of ACEIs and an increased risk of lung cancer (odds ratio [OR] 0.982, 95% confidence interval [CI], 0.873–1.104; *P* = 0.76). However, other researchers found that certain unsatisfactory inclusion criteria had been used and that inappropriate data had been included in those results. Since that publication, many original studies have been published, increasing the statistical power to further investigate the association between the use of ACEIs and the risk of lung cancer [19, 20].

Given the potential safety hazard to a large ACEIs treatment population, we aimed to perform a meta-analysis of studies in terms of the association between ACEIs use and the risk of lung cancer using strict scientific selection criteria. The findings of this meta-analysis are likely to be relevant for the long-term management of patients with chronic diseases receiving these drugs worldwide.

## Methods

This study was conducted in accordance with Preferred Reporting Items for Systematic Reviews and Meta-analyses (PRISMA) guidelines and was registered in the International Prospective Register of Systematic Reviews (PROSPERO; ID: CRD42022322228).

### Search strategy and selection criteria

This systematic review and meta-analysis included all publicly available data from PubMed, EMBASE, Cochrane Library, Ovid and Web of Science databases for studies published in English on lung cancer and ACEI clinical studies from inception to January 26, 2022.

According to the search strategy, ‘*angiotensin-converting enzyme inhibitor*’ and ‘*lung cancer*’ were used as search terms. Three authors (TKY, ZLW and ZCW) screened all related articles obtained from the databases.

The search terms were also used to search other databases (Google Scholar, Medline and CNKI), however, no additional results were obtained. Further details on the literature search strategy of each database can be found in the Supplementary Text. Three authors (TKY, ZLW and NW) performed a duplicate search independently, using reference management software (Endnote X9; Clarivate Analytics, London, UK). Duplicates were removed automatically.

Records were selected manually according to title, abstract, and full text. All English studies that met the following criteria were included: (i) the participants (aged ≥18) potentially needed ACEIs; (ii) the exposure/intervention was ACEIs; (iii) the control was other antihypertensive agents or placebo; (iv) studies in which adverse events after exposure were reported, and the outcome was lung cancer or the risk of lung cancer; (v) studies design was case–control, cohort or RCTs. Studies were excluded if: (i) the control drugs included any ACEIs; (ii) the median or mean follow-up period was <12 months; (iii) there were <100 participants in a study [21]; (iv) studies were published in non-English.

### Data extraction

Using an optimised extraction form, two authors (TKY and ZLW) retrieved pertinent data from the included studies separately, and another author (ZCW) undertook data monitoring and validation. Data on the study design, duration, baseline characteristics, follow-up period, adverse events and risk of lung cancer were retrieved. ORs and the morbidity of lung cancer were then calculated.

### Quality assessment

Publication bias was assessed using a funnel plot. Two authors (TKY and ZLW) assessed the risk of bias separately for the outcomes in the included studies using the Cochrane Risk of Bias 2 tool. The Newcastle–Ottawa Scale was also used to evaluate the quality of the included clinical studies. A final decision on any disagreement was made by all four authors (ZCW, NS, ML and BBL).

### Statistical analysis

Data analysis was performed using random-effects models. Cochrane Review Manager software (RevMan v5. 4. 1; The Nordic Cochrane Center) was used to compare the ORs of lung cancer in patients treated with or without ACEIs. The ORs with their 95% CIs for the morbidity of lung cancer were calculated. The specific population subgroup (patients with a history of smoking or DM) and a subgroup of patients from different regions were analysed. Statistical significance was set at *P* < 0.05. We used *I*^2^ and *chi*^2^ tests to assess heterogeneity. Low, moderate and high heterogeneity was defined as an *I*^2^ value of 25–50%; 51–75%; and >75%, respectively [22]. The chi-square test is used to assess heterogeneity and a *P* value of less than 0.1 for *chi*^*2*^ indicates the presence of statistical heterogeneity [22].

## Results

### Search results

**Fig. 1:**
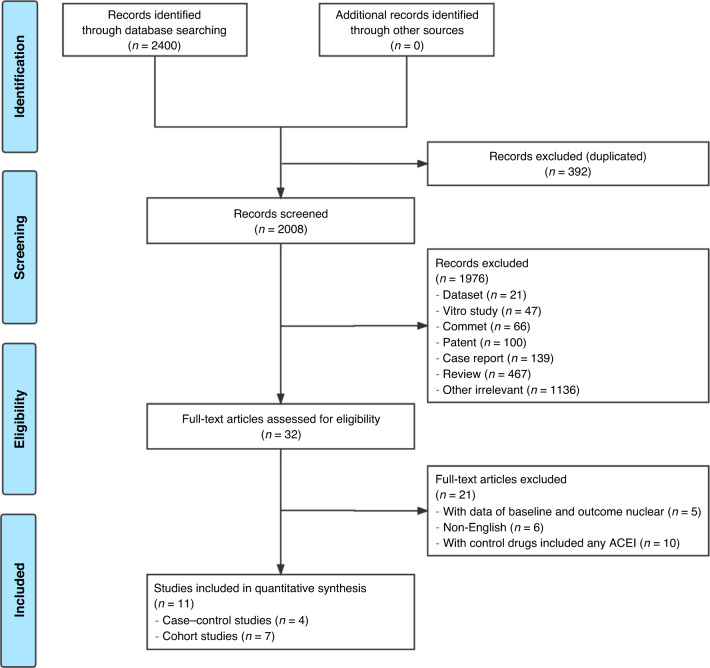
Flowchart. Flowchart outlining the literature selection for exploring the association between ACEI administration and the risk of lung cancer.

Based on the search strategy, 2400 records from the databases were screened, of which 392 duplicated records and 1976 records did not meet the inclusion criteria and were excluded. Finally, 11 clinical studies [12–14, 23–30], including seven cohort studies [12, 14, 23, 26–28, 30] and four case–control studies [13, 24, 25, 29], conducted between 1988 and 2020 were included in the meta-analysis (Fig. 1).

### Study characteristics

The 11 studies comprised 11 study groups and 13 control groups. Four groups were extracted from two cohort studies [26, 27] according to the different controls. The meta-analysis comprised a total of 13,061,226 patients, including 1,493,225 patients in the ACEI group and 11,568,001 patients in the non-ACEI group. These patients mainly resided in Asia [14, 27, 28], North America [23, 26], and Europe [12, 13, 24, 25, 30]. Patient and study characteristics of the cohort and case–control studies are described in Table 1. There was no significant imbalance in the included groups in terms of age and sex. All the studies indicated that patients with cancer prior to enrolment had been excluded. The study groups recorded the use of ACEIs, and the control groups recorded the use of angiotensin receptor blockers (ARBs) or other antihypertensive drugs.

**Table 1:** (**a**) Patient and study characteristics (Cohort studies); (**b**) patient and study characteristics (case–control studies).

a												
Study, year	Country	Study period	ACEI-exposed patients	Control patients	Reference group	Age (years)		Follow-up (years)	Men, %		History of cancer	ACEI category
						Exposed	Control		Exposed	Control		
Pasternak et al. [30]	Denmark	1998–2006	209,692	107,466	ARB	63	65	2.5	54.6	45.4	None	captopril, enalapril, lisinopril, perindopril, ramipril, quinapril, benazepril, fosinopril, trandolapril, and moexipril.
Hicks et al. [12]	UK	1988–2015	208,353	16,027	ARB	57.8	57.9	6.4	63.9	59.8	None	ramipril, lisinopril, perindopril
Jung et al. [27]	Korea	2002–2012	12,784^a^	281,178	ARB	60.3	56.8	6	64.6	54.9	None	NA
			5915^b^	185,199	ARB	59.9	56.4	6	65.1	55.2	None	NA
Lin et al. [14]	China	2000–2012	22,384^*^	22,384^*^	ARB	58.8	58.9	6.2	54.3	54.3	None	NA
Helgeson et al. [26]	USA	2000–2018	252,330^*^	252,330^*^	Neither^#^	58	59	9	42.5	44.6	None	NA
			90,103^*^	90,103^*^	ARB	61	61	9	50.3	51.2	None	NA
Anderson et al. [23]	USA	1996–2018	154,412	32,642	ARB	59.6	62.9	7.1	51.2	39.2	None	NA
Kumar et al. [28]	Pakistan	2005–2010	14,891	19,112	ARB	49	50	7.1	52.74	56.41	None	NA
**(b)**												
**Study, year**	**Countries**	**Study period**	**Cases/patients**	**Control patients**	**Ages (years)**		**Follow-up (years)**	**Men, %**		**History of cancer**	**ACEI category**	
					**Cases**	**Control**		**Cases**	**Control**	**History of cancer**		
Hallas et al. [25]	Denmark	2000–2005	16,343	65,281	65.5	65.5	7.8	47.7	47.7	None	NA	
Azoulay et al. [24]	UK	1995–2008	10,240	102,324	72.4	72.4	6.4	52.7	52.7	NA	NA	
Kristensen et al. [13]	Danish	2000–2015	9652	190,055	71.2	71.4	5.6	55.3	55.3	NA	NA	
Meng et al. [29]	Multicentre^c^	2004–2020	598	180,826	45–64 (28.0%)^d^	45–64 (28.1%)^d^	1.73**	47.8	49.9	None	benazepril, captopril, enalapril, fosinopril, lisinopril, moexipril, perindopril, quinapril, ramipril, trandolapril	

*ACEI* angiotensin-converting enzyme inhibitor, *ARB* angiotensin receptor blocker, *NA* not assessed, *UK* United Kingdom, *USA* United States of America.

^a^The entire cohort (prevalent user plus new user).

^b^The new-user cohort.

^*^Pairwise propensity score matching was done for the groups.

^#^Neither ACEIs nor ARBs.

^c^Multicentre: Mainly in the USA (67.5%), Canada (5.3%), UK (4.2%), Germany (3.4%) and others (19.6%).

^d^The age reported was stratified into different intervals, ‘45–64 (28.0%)’ meant that the patients aged 45–64 years accounted for 28.0%.

**Follow-up data from partially patients in FARS.

### Meta-analysis of ACEIs and risk of lung cancer

**Fig. 2:**
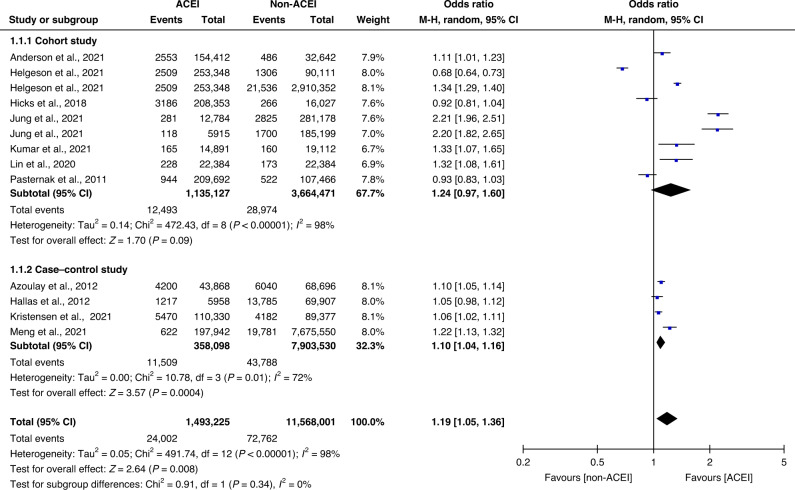
Subgroup analysis according to study design. Subgroup analysis of the study design in 11 studies concerning patients who did and did not receive ACEIs. Different size indicators are proportionate to study size and represent weights used in meta-analyses. The 95% CI of each study is indicated with horizontal lines; the diamond represents the pooled estimate with 95% CI.

In total, 96,764 of 13,061,226 patients had lung cancer outcomes. Overall, the pooled results of all the included studies showed that the use of ACEIs was associated with an increased risk of lung cancer (OR 1.19, 95% CI 1.05–1.36). A random-effects model was used to determine the ORs (the ORs for lung cancer risk in the nine cohort study datasets and the ORs for exposure to ACEIs in the four case–control study datasets) to eliminate possible bias in relation to the diverse research designs. Data from the cohort and case–control studies were used to create forest plots (Fig. 2). In the cohort studies, the risk of lung cancer in the study groups was comparable to that in the control groups (12,493 of 1,135,127 [1.10%] vs 28,974 of 3,664,471 [0.79%]; OR 1.24; 95% CI 0.97–1.60; *P* = 0.09; *I*^2^ = 98%; Supplementary Table B1). The ORs indicated that exposure to ACEIs was a risk factor in the case–control studies (11,509 of 358,098 [3.21%] vs 43,788 of 7,903,530 [0.55%]; OR 1.10; 95% CI 1.04–1.16; *P* = 0.0004; *I*^2^ = 72%; Supplementary Table B2). In addition, we calculated the incidence rate of lung cancer, which was 41,467 per 4,799,598 participants in all the cohort studies. The total incidence of lung cancer in the study groups using ACEIs was 1–3%.

The adoption of a random-effects model was justified due to the 98% heterogeneity in the overall study. The results of our meta-analysis remained robust irrespective of any methodological changes in sensitivity analyses. The results also remained unchanged when the risk of lung cancer was explored using a fixed effect model.

In addition, among the included studies, three studies [25, 27, 30] reported the occurrence of other cancers, such as hepatic cancer, gastric cancer, breast cancer, and prostate cancer, after using ACEIs (all ORs > 1, *P* < 0.05).

**Fig. 3:**
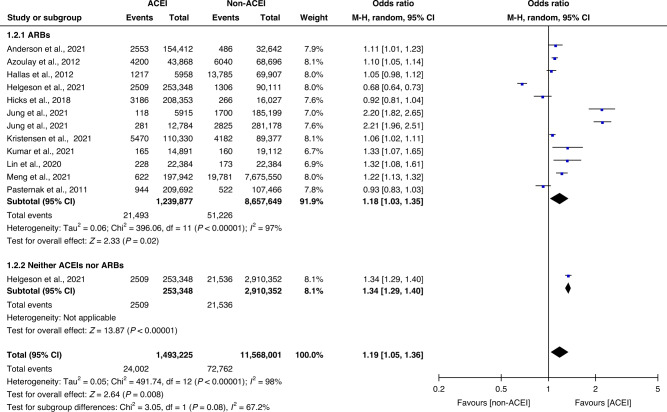
Subgroup analysis according to the reference group. Subgroup analysis of the reference group in 11 studies concerning patients who did and did not receive ACEIs. Different size indicators are proportionate to study size and represent weights used in meta-analyses. The 95% CI of each study is indicated with horizontal lines; the diamond represents the pooled estimate with 95% CI.

Considering possible confounders in the risk of lung cancer, we performed further subgroup analyses. The ACEI group was still considered the study group in all subgroup analyses. Compared with the two control subgroups—one [12–14, 23–30] comprising ARBs users (21,493 of 1,239,877 [1.73%] vs 51,226 of 8,657,649 [0.59%]; OR 1.18; 95% CI 1.03–1.35; *P* = 0.02; *I*^2^ = 97%) and the other [26] comprising patients neither with ACEIs nor ARBs (2509 of 253,348 [0.99%] vs 21,536 of 2,910,352 [0.74%]; OR 1.34; 95% CI 1.29–1.40), the ACEI group had a higher risk of lung cancer (Fig. 3).

**Fig. 4:**
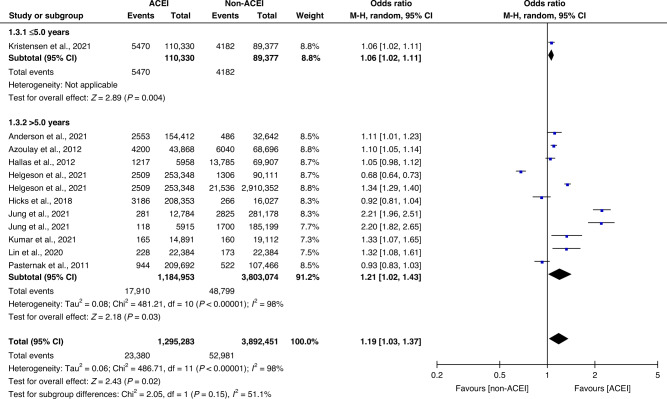
Subgroup analysis according to follow-up time. Subgroup analysis of the follow-up time in ten studies concerning patients who did and did not receive ACEIs. Different size indicators are proportionate to study size and represent weights used in meta-analyses. The 95% CI of each study is indicated with horizontal lines; the diamond represents the pooled estimate with 95% CI.

One study [29] did not voluntarily report mean follow-up time, based on a post hoc analysis of the database it used, we found that its mean follow-up time was 1.73 years, but the study was not included in the follow-up time subgroup analysis because some follow-up data were missing from the database. The other included studies in the subgroup analysis voluntarily reported the follow-up time of 3.6–9 years. Two subgroups separated by a mean follow-up period of five years were used to create forest plots. Although the use of ACEIs was not linked to a risk of lung cancer in the subgroup [13] with a mean follow-up period of ≤5.0 years (5470 of 110,330 [4.96%] vs 4182 of 89,377 [4.68%]; OR 1.06; 95% CI 1.02–1.11; *P* = 0.004), it was closely associated with the risk of lung cancer in the subgroup [12, 14, 23–28, 30] with a mean follow-up period of >5.0 years (17,910 of 1,184,593 [1.51%] vs 48,799 of 3,803,074 [1.28%]; OR 1.21; 95% CI 1.02–1.43; *P* = 0.03; *I*^2^ = 98%; Fig. 4).

Subgroup analyses of age and gender in patients with ACEIs taking were performed, non-elderly people (< 60-year-old) and women had high risk in ACEI-associated lung cancer. Specific population results are described in Supplementary Figs. C1 and C2. In most studies that described patients’ smoking history, the percentage of participants who smoked was usually >20.0%. There was a significant difference in the elevated incidence of lung cancer after using ACEIs both in the subgroup [23] with ≤20.0% smoking participants (2553 of 154,412 [1.65%] vs 486 of 32,642 [1.49%]; OR 1.11; 95% CI 1.01–1.23) and the subgroup [12, 24, 26–28] with >20.0% smoking participants (12,968 of 792,507 [1.64%] vs 33,833 of 3,570,675 [0.95%]; OR 1.28; 95% CI 1.00–1.64; *P* = 0.05; *I*^2^ = 99%), but the subgroup with >20.0% smoking participants showed a stronger association between the use of ACEIs and the risk of lung cancer (Supplementary Fig. C3).

Similarly, variations in the proportion of patients with DM affected the outcomes. The ORs for the exposure to ACEIs and lung cancer outcomes were not statistically significant in the subgroup [14, 25, 30] with ≤10.0% patients with DM (2389 of 238,034 [1.00%] vs 14,480 of 199,757 [7.25%]; OR 1.06; 95% CI 0.92–1.23; *P* = 0.94; *I*^2^ = 80%). However, the effect of the use of ACEIs on the risk of lung cancer was verified statistically in the subgroup [24, 26–28] with >10.0% of patients with DM (9782 of 584,154 [1.67%] vs 33,567 of 3,554,648 [0.94%]; OR 1.33; 95% CI 1.03–1.78; *P* = 0.03; *I*^2^ = 99%), suggesting a stronger association between the use of ACEIs and a higher risk of lung cancer in patients with DM (Supplementary Fig. C4). The ORs for the exposure to ACEIs and lung cancer outcomes were not statistically significant in the subgroup [14, 25, 26, 28] with ≤10.0% and >10.0% patients with CKD (*P* = 0.53) (Supplementary Fig. C5).

Four studies [12, 14, 23, 28] recorded a combination of medications (such as alpha-blockers, beta-blockers, calcium channel blockers, and diuretics). We further analysed the relationship between the use of ACEIs and the risk of lung cancer in the following three subgroups: unrecorded drug combinations, combinations with other oral antihypertensive medications, and combinations with statins. The subgroup with unrecorded drug combinations (17,870 of 1,093,185 [1.63%] vs 71,677 of 11,477,836 [0.62%]; OR 1.21; 95% CI 1.03–1.42; *P* = 0.02; *I*^2^ = 98%) had the same ORs for the risk of lung cancer as the subgroup with combinations with other oral antihypertensive medications (2946 of 191,687 [1.54%] vs 819 of 74,138 [1.10%]; OR 1.21; 95% CI 1.07–1.38; *P* = 0.004; *I*^2^ = 47%). However, the association was not apparent in one study [12] in which patients had used statins (3186 of 208,353 [1.53%] vs 266 of 16,027 [1.66%]; OR 0.92; 95% CI 0.81–1.04; *P* = 0.20; Supplementary Fig. C6).

### Study quality and risk of bias

All the studies were of high quality, scoring 7–9 on the Newcastle–Ottawa scale (Supplementary Table B3). The risk of bias in the included studies was assessed using the Cochrane Risk of Bias 2 tool. High selection bias was difficult to prevent because no RCTs were found. However, performance bias, detection bias, attrition bias, reporting bias, and other bias were found to be low risk (Supplementary Fig. C7). A funnel plot of the ORs showed no significant publication bias (Supplementary Fig. C8).

## Discussion

This systematic review and meta-analysis of seven cohort studies and four case–control studies with a total of 13,061,226 patients found a significant association between the use of ACEIs and an increased risk of lung cancer. Regardless of the type of cohort study subgroup or case–control study subgroup, there were similar tendencies. The incidence rate of lung cancer in patients using ACEIs was 1.61%; therefore, the lung cancer outcome could be classified as “common” in terms of adverse events following the use of ACEIs, according to the World Health Organization adverse drug reaction categorisation standards [31]. These results highlight the need for further research.

In a subgroup analysis based on the study design, on the one hand, the cohort study focused on ACEIs exposure in the study group and ARBs exposure in the control group, and the results showed no significant association between ACEIs administration and the risk of lung cancer (*P* = 0.09), compared with ARBs administration. However, it was proven that ARBs administration had an association with the risk of lung cancer [21]. In addition, according to epidemiological data from some reports, the population-based lung cancer incidence rates were 0.0409% (USA [32]), 0.05726% (China [33]), 0.0396% (Denmark [34]), 0.0348% (Canada [34]) and 0.00745% (India [34]). Whereas the mean lung cancer incidence rate in the cohort studies included in our study was 1.36% for patients taking ACEIs and 1.08% for patients taking ARBs, which allows for the inference that ACEIs and the risk of lung carcinogenesis are associated. On the other hand, the case–control study discussed the ORs of ACEIs exposure in lung cancer patients versus non-lung cancer patients, and the results were positive in this subgroup (*P* = 0.0004), so the association between ACEIs and lung cancer risk could be valid. The combined results of the cohort and case–control studies also generally confirmed the association between ACEIs and the risk of lung cancer.

Our results were contrary to those of a previous meta-analysis [18] that included 13 studies with 458,686 ACEI users, which reported no significant association between the use of ACEIs and the risk of lung cancer (relative risk, 0.982; 95% CI 0.873–1.104; *P* = 0.76). However, we found that some unsatisfactory inclusion criteria and inappropriate data had been included in the results of that meta-analysis. Another meta-analysis [35] that included RCTs also showed that there was no association between the use of ACEIs and the risk of cancer; however, some researchers [12] had suggested that the follow-up period of the included articles was not sufficiently long (median duration, 3.5 years). Many clinical studies have recently been published, increasing the statistical power and allowing for further investigation to determine the association between the use of ACEIs and the risk of lung cancer. Most of these studies have included patients from more extensive regions who had been followed up for an adequate amount of time.

Hicks [12] deemed that the incidence rate of lung cancer should be calculated using ‘person-years’, which has not been widely adopted in clinical studies of lung cancer, and concluded that the use of ACEIs might increase the risk of lung cancer. This evidence was sufficient to cast doubt on Bangalore’s [35] results. However, from our reanalysis of Hicks’ [12] data, we concluded that an insufficiently long study duration could explain Hicks’ findings. Data from Hicks’s [12] study otherwise showed no statistically significant difference between the use of ACEIs and an increased risk of lung cancer. Considering a long latent period with a continuous stimulation of harmful factors in lung cancer development, we set the subgroup analysis according to a 5-year follow-up period. The results of the analysis indicated that continuous treatment with ACEIs for >5 years might increase the risk of lung cancer.

Subgroup analyses of age and gender in patients using ACEIs were performed, non-elderly people and women had a high risk in ACEI-associated lung cancer. Although there is no direct literature evidence to support a higher risk of lung cancer in non-elderly people taking ACEIs, there is a study [36] that demonstrate that the risk factor for cough is female, which may provide a clue to explain the high risk of ACEI-related lung cancer in women.

Most patients with hypertension had been treated with multiple antihypertensive drugs, but the researchers had not recorded patient histories in detail. Compared with patients treated using drugs neither ACEIs nor ARBs, those treated with ACEIs had a higher risk of lung cancer. The potential influence of renin–angiotensin–aldosterone system (RAAS) inhibitors on carcinogenesis has been widely debated [12, 37–42]. A correlation between ARBs and the risk of lung cancer has been reported [21]. However, our results showed that the use of ACEIs was associated with a higher risk of lung cancer than the use of ARBs. Most of the clinical studies indicated that ACEIs had a major role in cancer development. More attention needs to be given to the correlation between the use of RAAS inhibitors and the risk of lung cancer in clinical practice.

Furthermore, compared with patients treated with statins, those treated with ACEIs combined with statins did not have an increased risk of lung cancer. Some studies [43–46] have deemed statins to have independent protective associations with the risk of lung cancer. Therefore, combinations of different ACEIs with statins should be investigated to monitor lung cancer morbidity and mortality.

We also analysed specific populations in the subgroups. Smoking can lead to lung cancer development. Patients with a history of smoking, some of whom were receiving ACEIs, had a higher risk of lung cancer than those who did not smoke. Moreover, patients treated with ACEIs in the DM subgroup might have had a higher risk of lung cancer than those in the non-DM subgroup. Although these findings suggest that patients with DM should avoid using ACEIs, clinical decision-making should be based on weighing the advantages and disadvantages in real-world settings for specific patients.

Many drug clinical trials may have assumed that different ethnicities treated with drugs would have varying types or degrees of side effects [47, 48]. While mechanisms explaining this phenomenon remain unclear, the findings of this meta-analysis indicated that, compared with patients from America and Europe, there was a significant correlation between the use of ACEIs and the risk of lung cancer in patients from Asia (Supplementary Fig. C9). However, one recent study [49] showed that Asian patients with COVID-19 might benefit from the use of ACEIs in terms of prognosis and mortality. The reason for this phenomenon is not clear, and the data only represented different geographical locations and was not a true reflection of the diversity of the different ethnicities.

Through inhibiting the angiotensin-converting enzyme, ACEIs reduce the degradation of bradykinin and substance P [50–52], resulting in their accumulation in the lungs, stimulating tumour cell proliferation, increasing vascular permeability, and contributing to tumour cell metastasis [28, 53–55]. Although our results suggest an association between the use of ACEIs and the risk of lung cancer, the underlying mechanism remains unclear.

ACEIs are widely used as first-line prescription drugs for patients with hypertension and congestive heart failure, especially when combined with CKD or DM [56–58]. Based on our results, for ACEI-related lung cancer risk, influencing factors included age, gender, ethnicity, duration of follow-up, smoking history, history of diabetes and drug combinations. Also, differences in clinical study design may affect the results to some extent. Given the widespread use of ACEIs, it is recommended that clinicians weigh up the various options when prescribing to make the most patient-friendly choice. This important finding provides high-quality evidence on the safety and occurrence of adverse events in the long-term management of patients with chronic diseases receiving these drugs worldwide.

This study had several limitations. First, we did not discuss the varying effects produced by different types of ACEIs due to different chemical formulae. Second, due to study design limitations, cohort studies and case–control studies are both susceptible to potential confounders. Unlike a previous meta-analysis of ARBs and cancer risk, this study did not include RCTs, although these have mainly evaluated the effect of ACEIs on cardiovascular and renal endpoints, without including tumour risk in the endpoints. Third, the included studies seldom described the pathological type of lung cancer, which might have been more informative for mechanistic studies. Furthermore, while publication bias cannot be readily ruled out, this meta-analysis provides a high level of evidence in support of the correlation between the use of ACEIs and the risk of lung cancer. In future, large multicentre RCTs are needed to corroborate our study findings.

## Conclusion

In summary, the findings of this systematic review and meta-analysis of 11 studies showed that the use of ACEIs is a relevant factor for lung carcinogenesis and that ACEIs use carries a greater risk than the use of ARBs, especially in patients of Asian ethnicity. The findings provide medication guidance on the long-term management of patients with chronic diseases who are treated with ACEIs worldwide. More RCTs are needed in the future to confirm the causal association between the use of ACEIs and the risk of lung cancer.

## Supplementary information

Supplementary Information

## Data Availability

The protocol of the study is available on PROSPERO, and the datasets used and analysed in this study are available in the manuscript and Supplementary Information. All authors had full access to all the data in the study and took responsibility for the accuracy of the data analysis.
