# Supplementary material for: Association between angiotensin-converting enzyme inhibitors and the risk of lung cancer: a systematic review and meta-analysis
Source: Br J Cancer. 2022 Nov 17;128(2):168–76. doi: 10.1038/s41416-022-02029-5 (PMC9670057; doi:10.1038/s41416-022-02029-5)
Supplement: Supplementary file 1 — Supplementary Information [file 41416_2022_2029_MOESM1_ESM.docx]

**Supplementary Information**

**[Appendix A: Supplementary Text 2](#_Toc16548)**

[eMethod 1. Search strategy in Web of Science 2](#_Toc15980)

[eMethod 2. Search strategy in Embase 2](#_Toc3626)

[eMethod 3. Search strategy in PubMed 3](#_Toc16417)

[eMethod 4. Search strategy in Cochrane 4](#_Toc4438)

[eMethod 5. Search strategy in Ovid 5](#_Toc18528)

**[Appendix B: Supplementary Table 6](#_Toc17265)**

[Table B1. Summary of lung cancer data from included cohort studies 6](#_Toc20763)

[Table B2. Summary of lung cancer data from included case-control studies 7](#_Toc19767)

[Table B3. Quality assessment of included clinical trials using the Newcastle-Ottawa Scale 8](#_Toc17542)

**[Appendix C: Supplementary Figure 9](#_Toc19170)**

[Figure C1. Subgroup analysis according to the mean age of patients 9](#_Toc9570)

[Figure C2. Subgroup analysis according to the female proportion of patients 10](#_Toc30171)

[Figure C3. Subgroup analysis according to the rate of patients with smoking habits 11](#_Toc10869)

[Figure C4. Subgroup analysis according to the rate of patients with diabetes mellitus 12](#_Toc2246)

[Figure C5. Subgroup analysis according to the rate of patients with chronic kidney disease 13](#_Toc28391)

[Figure C6. Subgroup analysis according to combination 14](#_Toc6391)

[Figure C7. Cochrane methodological quality assessment (Risk of Bias 2 Tool) of included studies (a) Risk of bias graph (b) Risk of bias summary 15](#_Toc9821)

[Figure C8. Funnel plot for assessing publication bias for lung cancer occurrence 16](#_Toc14491)

[Figure C9. Subgroup analysis according to the ethnicity of patients 17](#_Toc31490)

**[eReferences 18](#_Toc3862)**

**Appendix A: Supplementary Text**

**eMethod 1.** Search strategy in Web of Science

1. TS=(Kininase Il Antagonist OR Angiotensin Converting Enzyme Antagonist OR Kininase ll Inhibitor OR Angiotensin Converting Enzyme Inhibitor OR ACE Inhibitor OR Angiotensin I Converting Enzyme Inhibitor OR ACEI OR benazepril OR captopril OR cilazapril OR enalapril OR fosinopril OR perindopril OR lisinopril OR quinapril OR ramipril OR teprotide)
2. TS=(lung neoplasm OR pulmonary neoplasm OR lung cancer OR pulmonary cancer OR lung carcinoma)
3. 2 AND 1

**eMethod 2.** Search strategy in Embase

| No. | Query |
| --- | --- |
| 1 | 'dipeptidyl carboxypeptidase inhibitor'/exp |
| 2 | 'kininase ii antagonist':ab,ti |
| 3 | 'angiotensin converting enzyme antagonis':ab,ti |
| 4 | 'kininase ii inhibitor':ab,ti |
| 5 | 'angiotensin converting enzyme inhibitor':ab,ti |
| 6 | 'ace inhibitor':ab,ti |
| 7 | 'angiotensin i converting enzyme inhibitor':ab,ti |
| 8 | 'acei':ab,ti |
| 9 | 'benazepril':ab,ti |
| 10 | 'captopril':ab,ti |
| 11 | 'cilazapril':ab,ti |
| 12 | 'enalapri':ab,ti |
| 13 | 'fosinopril':ab,ti |
| 14 | 'perindopril':ab,ti |
| 15 | 'lisinopril':ab,ti |
| 16 | 'quinapril':ab,ti |
| 17 | 'ramipril':ab,ti |
| 18 | 'teprotide':ab,ti |
| 19 | 1 OR 2 OR 3 OR 4 OR 5 OR 6 OR 7 OR 8 OR 9 OR 10 OR 11 OR 12 OR 13 OR 14 OR 15 OR 16 OR 17 OR 18 |
| 20 | 'lung cancer'/exp |
| 21 | 'lung neoplasm':ab,ti |
| 22 | 'pulmonary neoplasm':ab,ti |
| 23 | 'pulmonary cancer':ab,ti |
| 24 | 'lung carcinoma':ab,ti |
| 25 | 20 OR 21 OR 22 OR 23 OR 24 |
| 26 | 19 AND 25 |

**eMethod 3.** Search strategy in PubMed

1. Angiotensin-Converting Enzyme Inhibitors[Mesh]
2. Kininase II Antagonist[Title/Abstract]
3. Angiotensin Converting Enzyme Antagonist[Title/Abstract]
4. Kininase II Inhibitor[Title/Abstract]
5. ACE Inhibitor[Title/Abstract]
6. Angiotensin I Converting Enzyme Inhibitor[Title/Abstract]
7. ACEI[Title/Abstract]
8. benazepril[Title/Abstract]
9. captopril[Title/Abstract]
10. cilazapril[Title/Abstract]
11. enalapril[Title/Abstract]
12. fosinopril[Title/Abstract]
13. perindopril[Title/Abstract]
14. lisinopril[Title/Abstract]
15. quinapril[Title/Abstract]
16. ramipril[Title/Abstract]
17. teprotide[Title/Abstract]
18. Angiotensin Converting Enzyme Inhibitor[Title/Abstract]
19. 1 OR 2 OR 3 OR 4 OR 5 OR 6 OR 7 OR 8 OR 9 OR 10 OR 11 OR 12 OR 13 OR 14 OR 15 OR 16 OR 17 OR 18
20. Lung Neoplasms[Mesh]
21. lung neoplasm[Title/Abstract]
22. pulmonary neoplasm[Title/Abstract]
23. lung cancer[Title/Abstract]
24. pulmonary cancer[Title/Abstract]
25. lung carcinoma[Title/Abstract])
26. 20 OR 21 OR 22 OR 23 OR 24 OR 25
27. 19 AND 26

**eMethod 4.** Search strategy in Cochrane

1. MeSH descriptor: [Lung Neoplasms] explode all trees
2. (lung neoplasm);ti,ab,kw OR(pulmonary neoplasm);ti,abkw OR (lung cancer);ti,ab,w OR(pulmonary cancer);ti,ab,kw OR(lung carcinoma);ti,ab,kw
3. 1 OR 2
4. MeSH descriptor: Angiotensin-Converting Enzyme Inhibitors] explode alltrees
5. (Kininase Ⅱ Antagonist):t,ab,kw OR(Angiotensin Converting Enzyme Antagonist):ti,ab,kw OR (Angiotensin Converting Enzyme Inhibitor):ti,ab,kw OR(ACE Inhibitor):ti,ab,kw AND(Angiotensin I Converting Enzyme Inhibitor):tiab,kw
6. (ACEI):ti,ab,kw OR(benazepril):tiab,kw OR (captopril):ti,ab,kw OR (cilazapril)ti,ab,kw AND (enalapri):ti,ab,kw
7. (fosinopril):ti,ab,kw OR(perindopri):ti,ab,kw OR(lisinopril):ti, ab,kw OR(quinapril):ti,abkw AND(ramipril):tiab,kw
8. (teprotide):ti,ab,kw
9. 4 OR 5 OR 6 OR 7 OR 8

10. 3 AND 9

**eMethod 5.** Search strategy in Ovid

1. lung cancer.ab,ti.
2. lung neoplasm.ab,ti.
3. pulmonary neoplasm.ab,ti.
4. pulmonary cancer.ab,ti.
5. lung carcinoma.ab,ti.
6. 1 OR 2 OR 3 OR 4 OR 5
7. Kininase II Antagonist.ab,ti.
8. Angiotensin Converting Enzyme Antagonist.ab,ti.
9. Kininase II Inhibitor.ab,ti.
10. Angiotensin Converting Enzyme Inhibitor.ab,ti.
11. ACE Inhibitor.ab,ti.
12. Angiotensin I Converting Enzyme Inhibitor.ab,ti.
13. ACEI.ab,ti.
14. benazepril.ab,ti.
15. captopril.ab,ti.
16. cilazapril.ab,ti.
17. enalapril.ab,ti.
18. fosinopril.ab,ti.
19. perindopril.ab,ti.
20. lisinopril.ab,ti.
21. quinapril.ab,ti.
22. ramipril.ab,ti.
23. teprotide.ab,ti.
24. 7 OR 8 OR 9 OR 10 OR 11 OR 12 OR 13 OR 14 OR 15 OR 16 OR 17 OR 18 OR 19 OR 20 OR 21 OR 22 OR 23
25. 6 AND 24

**Appendix B: Supplementary Table**

**Table B1.** Summary of lung cancer data from included cohort studies

| **Study, (year)** | **Lung cancer events（ACEI Exposed)** | **Total（Exposed)** | **Incidence rate of lung cancer with ACEI exposed** | **Lung cancer events (ARB control)** | **Total（Control)** | **Incidence rate of lung cancer with ARB control** |
| --- | --- | --- | --- | --- | --- | --- |
| Pasternak,(2011)^1^ | 944 | 209692 | 0.45% | 522 | 107466 | 0.49% |
| Hicks,(2018)^2^ | 3186 | 208353 | 1.53% | 266 | 16027 | 1.66% |
| Jung,(2021)^3^ | 281 | 12784 | 2.20%^a^ | 2825 | 281178 | 1.00% |
|  | 118 | 5915 | 1.99%^b^ | 1700 | 185199 | 0.92% |
| Lin,(2020)^4^ | 228 | 22384 | 1.02% | 173 | 22384 | 0.77% |
| Helgeson,(2021)^5^* | 2509 | 253348 | 0.99% | 1306 | 90111 | 1.45% |
| Anderson,(2021)^6^ | 2553 | 154412 | 1.65% | 486 | 32642 | 1.49% |
| Kumar,(2021)^7^ | 165 | 14891 | 1.11% | 160 | 19112 | 0.84% |

Abbreviation: ACEI, Angiotensin-Converting Enzyme Inhibitor; ARB, Angiotensin-Receptor Blocker.

Note:

^a^ the entire cohort (prevalent user plus new-user).

^b^ the new-user cohort.

* Incidence rate of lung cancer with neither ACEI/ARB was 0.74% (lung cancer 21536/ patients of neither 2910352).

**Table B2.** Summary of lung cancer data from included case-control studies

| **Study, (year)** | **Lung cancer events in all patients** | **ACEI exposed patients with lung cancer events** | **Without lung cancer events in all patients** | **ACEI exposed patients without lung cancer events** | **OR(95%Cl)** |
| --- | --- | --- | --- | --- | --- |
| Azoulay,(2012)^8^ | 10240 | 4200 | 102324 | 39668 | 1.10(1.05,1.14) |
| Hallas,(2012)^9^ | 16343 | 1217 | 65281 | 4741 | 1.05(0.98.1.12) |
| Kristensen,(2021)^10^ | 9652 | 5470 | 190055 | 104860 | 1.06(1.02,1.11) |
| Meng,(2021)^11^ | 20403 | 622 | 7853089 | 197320 | 1.22(1.13,1.32) |

Abbreviation: OR, odds ratio.

**Table B3.** Quality assessment of included clinical trials using the Newcastle-Ottawa Scale

| **Study, (year)** | **Countries** | **Study design** | **Study quality** |
| --- | --- | --- | --- |
| Hallas,(2012)^9^ | Denmark | case-control | 7 |
| Azoulay,(2012)^8^ | UK | case-control | 8 |
| Kristensen,(2021)^10^ | Danish | case-control | 7 |
| Meng,(2021)^11^ | Multi-center | case-control | 7 |
| Pasternak,(2011)^1^ | Denmark | cohort | 8 |
| Hicks,(2018)^2^ | UK | cohort | 8 |
| Jung,(2021)^3^ | Korea | cohort | 8 |
| Lin,(2020)^4^ | China | cohort | 7 |
| Helgeson,(2021)^5^ | USA | cohort | 8 |
| Anderson,(2021)^6^ | USA | cohort | 9 |
| Kumar,(2021)^7^ | Pakistan | cohort | 8 |

**Appendix C: Supplementary Figure**

**Figure C1.** Subgroup analysis according to the mean age of patients

Note: Subgroup analysis of the mean age of patients in 11 studies of patients who did and did not receive ACEIs. Different size indicators are proportionate to study size and represent weights used in meta-analyses. The 95% Cl of each study are indicated by horizontal lines; the diamond represents the pooled estimate with 95% Cl.

**Figure C2.** Subgroup analysis according to the female proportion of patients

Note: Subgroup analysis of the female proportion in 11 studies of patients who did and did not receive ACEIs. Different size indicators are proportionate to study size and represent weights used in meta-analyses. The 95% Cl of each study are indicated by horizontal lines; the diamond represents the pooled estimate with 95% Cl.

**Figure C3.** Subgroup analysis according to the rate of patients with smoking habits

Note: Subgroup analysis of the smoking rate in 6 studies of patients who did and did not receive ACEIs. Different size indicators are proportionate to study size and represent weights used in meta-analyses. The 95% Cl of each study are indicated by horizontal lines; the diamond represents the pooled estimate with 95% Cl.

**Figure C4.** Subgroup analysis according to the rate of patients with diabetes mellitus

Note: Subgroup analysis of the diabetes rate in 7 studies of patients who did and did not receive ACEIs. Different size indicators are proportionate to study size and represent weights used in meta-analyses. The 95% Cl of each study are indicated by horizontal lines; the diamond represents the pooled estimate with 95% Cl.

**Figure C5.** Subgroup analysis according to the rate of patients with chronic kidney disease

Note: Subgroup analysis of the proportion of patients with CKD in 4 studies of patients who did and did not receive ACEIs. Different size indicators are proportionate to study size and represent weights used in meta-analyses. The 95% Cl of each study are indicated by horizontal lines; the diamond represents the pooled estimate with 95% Cl.

**Figure C6.** Subgroup analysis according to combination

Note: Subgroup analysis of the drug combination in 11 studies of patients who did and did not receive ACEIs. Different size indicators are proportionate to study size and represent weights used in meta-analyses. The 95% Cl of each study are indicated by horizontal lines; the diamond represents the pooled estimate with 95% Cl.

**Figure C7.** Cochrane methodological quality assessment (Risk of Bias 2 Tool) of included studies (a) Risk of bias graph (b) Risk of bias summary


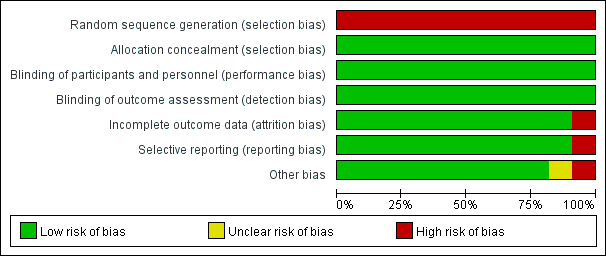


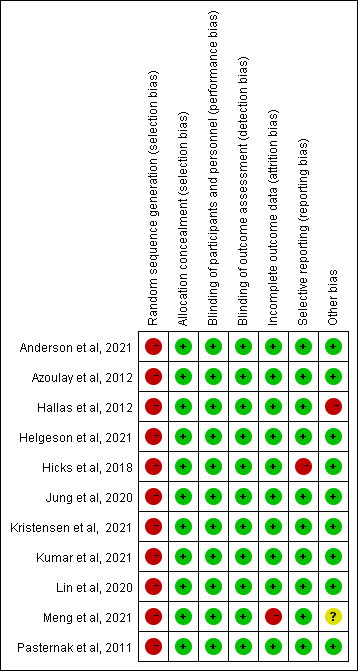


**Figure C8.** Funnel plot for assessing publication bias for lung cancer occurrence

**Figure C9.** Subgroup analysis according to the ethnicity of patients

Note: Subgroup analysis of the study region in 11 studies of patients who did and did not receive ACEIs. Different size indicators are proportionate to study size and represent weights used in meta-analyses. The 95% Cl of each study are indicated by horizontal lines; the diamond represents the pooled estimate with 95% Cl.

**eReferences**

1 Pasternak, B., Svanström, H., Callréus, T., Melbye, M. & Hviid, A. Use of angiotensin receptor blockers and the risk of cancer. *Circulation* **123**, 1729-1736 (2011).

2 Hicks, B. M. *et al.* Angiotensin converting enzyme inhibitors and risk of lung cancer: population based cohort study. *BMJ (Clinical research ed.)* **363**, k4209 (2018).

3 Jung, M. H. *et al.* Effect of angiotensin receptor blockers on the development of cancer: A nationwide cohort study in korea. *Journal of clinical hypertension (Greenwich, Conn.)* **23**, 879-887 (2021).

4 Lin, S. Y. *et al.* Association between Angiotensin-Converting Enzyme Inhibitors and Lung Cancer-A Nationwide, Population-Based, Propensity Score-Matched Cohort Study. *Cancers* **12**, 747 (2020).

5 Helgeson, S. A. *et al.* Association between Angiotensin-Converting Enzyme Inhibitors and Angiotensin Receptor Blockers and Lung Cancer. *Southern medical journal* **114**, 607-613 (2021).

6 Anderson, J. L. *et al.* Evaluation of TReatment With Angiotensin Converting Enzyme Inhibitors and the Risk of Lung Cancer: ERACER—An Observational Cohort Study. **26**, 321-327 (2021).

7 Kumar, P. *et al.* Comparison Between Angiotensin-Converting Enzyme Inhibitors and Angiotensin Receptor Blockers for Incidence of Lung Cancer: A Retrospective Study. *Cureus* **13**, e14788 (2021).

8 Azoulay, L. *et al.* Long-term use of angiotensin receptor blockers and the risk of cancer. *PloS one* **7**, e50893 (2012).

9 Hallas, J., Christensen, R., Andersen, M., Friis, S. & Bjerrum, L. Long term use of drugs affecting the renin-angiotensin system and the risk of cancer: a population-based case-control study. *British journal of clinical pharmacology* **74**, 180-188 (2012).

10 Kristensen, K. B., Hicks, B., Azoulay, L. & Pottegård, A. Use of ACE (Angiotensin-Converting Enzyme) Inhibitors and Risk of Lung Cancer: A Nationwide Nested Case-Control Study. *Circulation. Cardiovascular quality and outcomes* **14**, e006687 (2021).

11 Meng, L. *et al.* Lung Cancer Adverse Events Reports for Angiotensin-Converting Enzyme Inhibitors: Data Mining of the FDA Adverse Event Reporting System Database. *Frontiers in medicine* **8**, 594043 (2021).
